# Successful Low-Cost Scaffold-Free Cartilage Tissue Engineering Using Human Cartilage Progenitor Cell Spheroids Formed by Micromolded Nonadhesive Hydrogel

**DOI:** 10.1155/2017/7053465

**Published:** 2017-12-20

**Authors:** Mellannie P. Stuart, Renata A. M. Matsui, Matheus F. S. Santos, Isis Côrtes, Mayra S. Azevedo, Karina R. Silva, Anderson Beatrici, Paulo Emílio C. Leite, Priscila Falagan-Lotsch, José M. Granjeiro, Vladimir Mironov, Leandra S. Baptista

**Affiliations:** ^1^Laboratório de Bioengenharia Tecidual, Instituto Nacional de Metrologia, Qualidade e Tecnologia (Inmetro), Duque de Caxias, RJ, Brazil; ^2^Programa de Pós-graduação em Biotecnologia, Instituto Nacional de Metrologia, Qualidade e Tecnologia (Inmetro), Duque de Caxias, RJ, Brazil; ^3^Programa de Pós-graduação em Biomedicina Translacional, Universidade do Grande Rio (UNIGRANRIO), Duque de Caxias, RJ, Brazil; ^4^Núcleo Multidisciplinar de Pesquisa em Biologia (Numpex-Bio), Universidade Federal do Rio de Janeiro (UFRJ) Polo de Xerém, Duque de Caxias, RJ, Brazil; ^5^Department of Chemistry, University of Illinois at Urbana–Champaign, Urbana, IL 61801, USA; ^6^Escola de Odontologia, Universidade Federal Fluminense (UFF), Niterói, RJ, Brazil; ^7^Regenerative Medicine Center, Sechenov Medical University, Moscow, Russia

## Abstract

The scaffold-free tissue engineering using spheroids is pointed out as an approach for optimizing the delivery system of cartilage construct. In this study, we aimed to evaluate the micromolded nonadhesive hydrogel (MicroTissues®) for spheroid compaction (2-day culture) and spontaneous chondrogenesis (21-day culture) using cartilage progenitors cells (CPCs) from human nasal septum without chondrogenic stimulus. CPC spheroids showed diameter stability (486 *μ*m ± 65), high percentage of viable cells (88.1 ± 2.1), and low percentage of apoptotic cells (2.3%). After spheroid compaction, the synthesis of TGF-*β*1, TGF-*β*2, and TGF-*β*3 was significantly higher compared to monolayer (*p* < 0.005). Biomechanical assay revealed that the maximum forces applied to spheroids after chondrogenesis were 2.6 times higher than for those cultured for 2 days. After spontaneous chondrogenesis, CPC spheroids were entirely positive for N-cadherin, collagen type II and type VI, and aggrecan and chondroitin sulfate. Comparing to monolayer, the expression of SOX5 and SOX6 genes analyzed by qPCR was significantly upregulated (*p* < 0.01). Finally, we observed the capacity of CPC spheroids starting to fuse. To the best of our knowledge, this is the first time in the scientific literature that human CPC spheroids were formed by micromolded nonadhesive hydrogel, achieving a successful scaffold-free cartilage engineering without chondrogenic stimulus (low cost).

## 1. Introduction

The classical tissue engineering relies on scaffold-based approaches in which the scaffold serves as a substitute for extracellular matrix. Despite the reasonable success reached by classical tissue engineering over the past decades [1], some remarkable issues are still unsolved, especially that related to the optimal delivery system for a better retention and integration with surrounding tissue. As an alternative, 3D tissue constructs can be produced in the absence of scaffolds [2]. This strategy is named scaffold-free tissue engineering, pointed out as having superior results since cells are responsible for synthesizing their own extracellular matrix optimizing cell-matrix and cell-cell interactions, recreating their native tissue microenvironment and recapitulating tissue morphogenesis [3]. Furthermore, scaffold-free 3D constructs have showed long-term retention in the implantation site [4].

The pellet culture, hanging-drop, and 96-well plate have been notably used for 3D cartilage constructs in scaffold-free tissue engineering [5]. In pellet culture, the cellular self-organization is responsible for cell aggregate formation by applying an external force. The main limitation of aggregates is related to their uncontrolled and nonhomogeneous shape [6]. On the other hand, the spheroids are morphologically different from aggregates especially due to their distinct dimensions and gross appearances related to size, thickness, and shape. In contrast to aggregates formed by external forces, spheroids are formed by self-assembling process using nonadherent hydrogel molds or platforms such as hanging-drop, 96-well plate, and recently micromolded nonadhesive hydrogels [7, 8]. The main advantage of recent platforms of micromolded nonadhesive hydrogel is seeding cell suspension with single pipetting, reducing substantially technical errors, and allowing automation [3].

The first step of cartilage development in developing embryo is mesenchymal cell condensation [9]. The morphogenic proteins of the transforming growth factor-beta (TGF-beta) superfamily initiate the condensation phase by increasing expression of N-cadherins. In fact, this phase is mimic by cell-driven spheroid formation (self-assembling process), since cell-cell interaction is mediated by N-cadherins leading to spheroid compaction [10]. On the other hand, hydrogels, commonly used in scaffold-based cartilage tissue engineering, are responsible to impair cell-cell interaction [11]. Also at condensation phase, the SOX5 and SOX6 proteins form a complex with SOX9 in a close cooperation to regulate positively the col2a1 (collagen type 2a1) gene expression [12]. Thus, extracellular matrix-specific proteins such as collagen type II as well as aggrecan appear concomitant with condensation [13].

Several studies have been published using scaffold-free approaches for cartilage tissue engineering [10, 14], most of them using cell aggregates instead of spheroids [15]. Besides issues about their uncontrolled and nonhomogeneous shape as mentioned before, the 3D cell culture techniques commonly applied for aggregate formation are susceptible to external forces, jeopardizing the condensation phase [2]. In respect to stem/progenitor cell human source, although mesenchymal stem/stromal cells show chondrogenic capacity, they usually progress to hypertrophic chondrocyte phenotype [16]. A population of progenitor cells dwelling on the surface of articular cartilage has been described by several research groups. This source of cells shares similarities with mesenchymal stem/stromal cells mainly related to surface marker expression and multipotentiality [17]. Besides articular cartilage, it is also possible to isolate progenitor cells in cartilage from human nasal septum [18].

In this study, we aimed to evaluate the micromolded nonadhesive hydrogel for cell spheroid formation and compaction with a homogeneous size and shape and subsequent spontaneous chondrogenesis using cartilage progenitor cells (CPCs) from human nasal septum, firstly described by our research group [19]. The spontaneous chondrogenic capacity of CPCs was already proven in 3D culture system [19, 20]. Our analysis points refer to spheroid compaction (2-day culture) and spontaneous chondrogenesis (21-day culture). From our knowledge, this is the first time in scientific literature that micromolded nonadhesive hydrogel is tested for cartilage tissue engineering. Furthermore, our source of CPCs is advantageous since the cell culture medium for chondrogenic induction is totally free of induction factors such as TGF-*β*1 or TGF-*β*3 [20] substantially reducing the costs for an optimized delivery system using spheroids.

## 2. Material and Methods

### 2.1. Human Cartilage Sampling

Cartilage fragments from nasal septum were obtained from healthy donors (*n* = 3, age from 18 to 40 years) who underwent plastic surgery. This study was approved by the Research Ethics Committee of the Clementino Fraga Filho University Hospital, Federal University of Rio de Janeiro, Brazil (Protocol 145-09), and informed consent was obtained from all donors included in the study. The samples were stored at 4°C after the surgery, and the cartilage progenitor cell isolation was performed within 18 hours.

### 2.2. Isolation and *In Vitro* Expansion of Cartilage Progenitor Cells (CPCs)

CPCs were isolated as previously described [19]. Briefly, cartilage fragments from nasal septum were incubated with collagenase IA 1 mg/ml for 1 hour. Cells were harvested by centrifugation and plated in culture flasks. The culture flasks were maintained at 37°C in a humid atmosphere with 5% carbon dioxide with low-glucose Dulbecco's modified Eagle's medium (DMEM; Sigma-Aldrich) supplemented with 10% fetal bovine serum (FBS; GIBCO), 100 U/ml penicillin and 100 *μ*g/ml streptomycin (Sigma-Aldrich). The medium was changed every 3 to 5 days until the cell monolayer reached confluence. At confluence, the cells were harvested with 0.125% trypsin (GIBCO) and 0.78 mM ethylenediaminetetraacetic acid (GIBCO).

### 2.3. Cartilage Progenitor Cell Spheroid Culture

**Figure 1:**
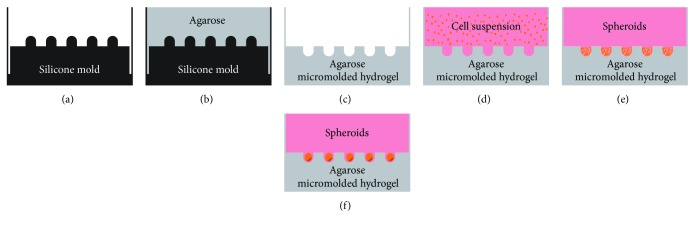
Scheme of cartilage progenitor cell spheroid culture. (a) Silicone molds were used from MicroTissues 3D Petri Dish. (b) The agarose solution was dispensed into silicone molds, resulting in the micromolded nonadhesive agarose hydrogel (c). (d) The cell suspension was carefully seeded into cell seeding chamber with single pipetting. (e) The cell suspension decanted into circular recesses after few minutes resulting in spheroids after 18 hours (f).

For spheroid culture, silicone molds were used in order to produce the micromolded nonadhesive agarose hydrogel (agarose 2%—Ultrapure Agarose, Invitrogen, Carlsbad, CA, USA—in NaCl 0.9% solution) with 300 *μ*m or 800 *μ*m diameter in each circular recesses (MicroTissues 3D Petri Dish, Sigma) according to manufacturer's protocol (Figure 1). For cell seeding, a suspension of 2 × 10^6^ cells was prepared in 190 ul of DMEM supplemented with 50 *μ*g/ml ascorbic acid (Sigma), 1.25 *μ*g/ml human albumin (FarmaBiagini SPA), 6.25 *μ*g/ml insulin (Novo Nordisk), 100 U/ml penicillin, and 100 *μ*g/ml streptomycin (Sigma) and Insulin-Transferrin-Selenium, ITS 1X (Lonza). Cell density was chosen according to recommendations of the silicone mold manufacturer (MicroTissues 3D Petri Dish, Sigma). The cell suspension was carefully seeded into the hydrogel-cell seeding chamber. The micromolded nonadhesive agarose hydrogel was maintained for at least one hour at 37°C in a humid atmosphere with 5% carbon dioxide for cell sedimentation inside circular recesses. After that, 2.5 ml of medium were added to the outside of the cell seeding chamber. The spheroids were formed after 18 hours and the spheroid culture was maintained at 37°C in a humid atmosphere with 5% carbon dioxide until 21 days. The medium was changed twice a week. All the analysis points refer to spheroid compaction (2-day culture) and spontaneous chondrogenesis (21-day culture).

### 2.4. Measurement of the Spheroid Diameter

The spheroids cultivated in micromolded nonadhesive agarose hydrogel with 300 *μ*m or 800 *μ*m diameter in each circular recesses were examined after 2-day culture and 21-day culture under an optical microscope (Leica DMI 6000 B) equipped with Leica DF 500 digital camera. The diameter of the spheroids was determined using AxioVision software (AxioVs40 V4.8.2.0) with the bar size of each image as a reference for the measurements, which were expressed in micrometers. Results were expressed as the mean ± standard error. A total of 45 spheroids from 2 or 3 micromolded nonadhesive hydrogels were measured randomly. Both assays were performed in triplicate from three different cell samples (*n* = 3).

### 2.5. Cell Viability and Apoptosis Analysis

For cell viability analysis, the spheroids cultivated in micromolded nonadhesive agarose hydrogel with 800 *μ*m diameter in each circular recesses were collected after spheroid compaction (2-day culture) and spontaneous chondrogenesis (21-day culture) and incubated with 1 mg/ml collagenase type I (Sigma Chemical), 0.125% trypsin (Gibco), and 0.78 mM ethylenediaminetetraacetic acid (Gibco) in a shaking water bath at 37°C for 40 minutes mixing well by pipetting up and down every 2-3 min [21]. After centrifugation, the cell suspension resulting from the spheroids was washed with phosphate-buffered saline and incubated for 30 minutes at 4°C with CD44-phycoerythrin (BD Biosciences). Subsequently, the cells were washed again with phosphate-buffered saline and incubated with 7-actinomycin D (7AAD, BD Biosciences, Franklin Lakes, NJ, USA) for 10 minutes. Cell apoptosis was evaluated by staining cells with Annexin V and PI (propidium iodide) according to the manufacturer's recommendations (BD Biosciences). For each tube, a total of 20,000 events of both samples were acquired in a FACSAria III (BD Biosciences). The flow cytometry analysis was performed using the software FACS Diva 8.0 (BD Biosciences). The viable cells identified by 7AAD exclusion were expressed as a percentage of total cells. The assays were performed from three different cell samples (*n* = 3).

### 2.6. Multiplex Analysis of Secreted Products

The culture medium was changed after spheroid compaction (2-day culture) and spontaneous chondrogenesis (21-day culture) in micromolded nonadhesive agarose hydrogel with 800 *μ*m diameter in each circular recesses. For the monolayer, the culture medium was changed after 2 days of cell culture. After 24 hours of culture medium change, the supernatant of all samples was collected and frozen at −80°C. The determination of proteins in the supernatant was carried out using the LuminexxMAP technology based on a magnetic bead panel for recognition of human TGF-*β*1, TGF-*β*2, and TGF-*β*3. The Bio-Plex Magpix apparatus (Biorad Laboratories Inc., Hercules, CA, USA) was calibrated and validated, reagents reconstituted, and standard curve prepared. Experimental procedures were preceded by washing steps with automated Bio-Plex Pro wash station (Biorad Laboratories Inc., Hercules, CA, USA). The concentration of each secreted product was quantified by the xPONENT software version 4.2 (Biorad Laboratories Inc., Hercules, CA, USA). Results were expressed as picograms per milliliter (*ρ*g/ml). The assay was performed from three different cell samples (*n* = 3) using three replicates from each sample.

### 2.7. Biomechanical Assay

Spheroid resistance to compression was evaluated using the Microsquisher (CellScale) equipment. Spheroids cultured for spheroid compaction (2-day culture) and spontaneous chondrogenesis (21-day culture) in micromolded nonadhesive agarose hydrogel with 800 *μ*m diameter in each circular recesses were collected and disposed in a platform inside a PBS bath at 37°C. A parallel plate exerted cycles of compression that consisted of a vertical force with amplitudes of 25% from spheroid diameter. Each cycle consisted of a load phase (20 s) followed by a recovery one (10 s). The maximum forces needed to reach 25% of spheroids compression were determined. Five cycles were performed in triplicate from three different cell samples (*n* = 3).

### 2.8. RNA Isolation and Quantification

After 21 days of cell culture, the monolayer and the spheroids (spontaneous chondrogenesis) cultivated in micromolded nonadhesive agarose hydrogel with 800 *μ*m diameter in each circular recesses were incubated with RLT buffer (Qiagen, Sweden). RNA extraction was performed with RNeasy Mini Kit according to manufacturer's instructions (Qiagen, Sweden). The protocol established for purification of total RNA from Animal Tissues was used to isolate the material for the spheroids. For the monolayer, the RNA was extracted from 1 × 10^6^ cells using the protocol established for animal cells. RNA integrity was evaluated by electrophoresis on a 1.5% denaturing agarose gel. The RNA purity and yield were determined by spectrophotometry (Nanodrop 2000C, Thermo Scientific) at 260 nm and 280 nm. Samples presenting A260/A280 ratio of ~2.0 were considered for gene expression analysis. The RNA isolation was performed from three different cell samples (*n* = 3).

### 2.9. Quantitative Real-Time PCR (qPCR)

The expression levels of SOX5, SOX6, and SOX9 genes, involved in the regulation of chondrogenesis, were evaluated in cells cultured as monolayer and as spheroids by quantitative polymerase chain reaction (qPCR). All reagents were purchased from Applied Biosystems, USA. The qPCR was performed using the AgPath-ID™ one-step RT-PCR kit. Briefly, 1 *μ*l of purified RNA (50 ng/*μ*l) was reverse transcribed and amplified in a 10 *μ*l reaction mixture containing 5 *μ*l of 2x RT-PCR buffer, 0.4 *μ*l of 25x RT-PCR enzyme mix, and 1.25 *μ*l yeast RNA 5 mg/ml (Ambion). Specific primers and TaqMan® probe sets for each gene were obtained from Assay-on-Demand Gene Expression Products (Applied Biosystems). The RNA samples were run in triplicate for each gene. CASC3 (cancer susceptibility candidate gene 3) was used as a reference gene to normalize the expression of target gene samples in both monolayer and spheroids. The thermal cycling conditions comprised 10 min RT step at 45°C, a 10 min initial PCR activation step at 95°C (AmpliTaq Gold activation) followed by 40 cycles of 95°C for 15 s, and 60°C for 45 s each (ABI 7500, Applied Biosystems). Relative expression levels of SOX genes were calculated for each sample after normalization against the average of the geometric mean of CASC3 gene (internal control) using the ΔΔCt method for comparing relative fold expression differences between cells cultured as monolayer and cells cultured as spheroids.

### 2.10. Histology and Immunohistochemistry

For histological preparations, the spheroids cultivated in nonadhesive agarose hydrogel with 800 *μ*m diameter in each circular recesses for 21 days (spontaneous chondrogenesis) were collected and fixed in 4% paraformaldehyde in phosphate buffered saline (PBS), pH 7.4, for 1 h at room temperature. After fixation, tissues were dehydrated in graded ethanol, cleared in xylol, and embedded in paraffin at 60°C. Sections of 5 *μ*m were prepared using the Slee Medical microtome (CUT 5062). Sections were collected onto 0.01% poly-L-lysine-coated slides (Sigma Chemica) and stained by hematoxylin and eosin (H/E).

For immunohistochemistry analysis, paraffin sections were collected onto silano-treated slides (Starfrost®). After dewaxing, sections were hydrated and incubated with the protein blocking solution for 15 min. Antigen unmasking was performed only for collagen type II using the enzyme chondroitinase ABC (Sigma Chemical) at 37°C for 30 min. Endogenous peroxidase was blocked using the peroxidase blocking solution for 15 min followed by PBS-Tween wash. The following primary antibodies (Abcam, Cambridge, UK) were incubated for 1 h at room temperature: collagen type II (1 : 50), collagen type VI (1 : 100), aggrecan (1 : 100), chondroitin sulfate (1 : 300), and N-cadherin (1 : 800). All reactions were done in the same moment. After washing in PBS-Tween, secondary antibody staining was performed using the Reveal-Polyvalent HRP kit (Code SPB 125, Spring). Peroxidase was revealed with diaminobenzidine (DAB liquid + substrate—chromogen system DAB-125, Spring®). Sections were then counterstained with hematoxylin, dehydrated, mounted in Entellan® (Merck), and examined under Leica DM-2500 optical microscope. Nonspecific binding of secondary antibody was monitored by carrying out the immune reaction in the absence of the primary antibody.

### 2.11. Fusion Assay

Spheroids cultured for spheroid compaction (2-day culture) and spontaneous chondrogenesis (21-day culture) in micromolded nonadhesive agarose hydrogel with 800 *μ*m diameter in each circular recesses were collected and transferred to 96-well flat bottom plates previously coated with agarose (agarose 2%—UltrapureAgarose, Invitrogen, Carlsbad, CA, USA—in NaCl 0.9% solution) in order to avoid adhesion of spheroids in well surface. Four spheroids were seeded per well in close contact and maintained in culture for until 7 days. Each well was examined under Zeiss 37,081 phase contrast microscope after 1 hour, 1 and 7 days.

### 2.12. Statistical Analysis

Nonparametric and unpaired Student's *t*-test (Mann–Whitney test) was performed to compare data concerning cell viability and mechanical tests of spheroids cultivated for 2 days with those cultivated for 21 days. One-way analysis of variance test was used for comparisons among the monolayer, spheroids cultivated for 2 days, and spheroids cultivated for 21 days in the TGF-*β* secretion assay. The results in the graphs are expressed as the mean ± standard deviation, and *p* values less than 0.05 were considered statistically significant. All analyses were performed using the software GraphPad Prism 6.0 (GraphPad Software, San Diego, CA, USA).

Statistical analysis of the qPCR data was performed using the web-based RT2 Profiler™ PCR Array Data Analysis software (SABiosciences, http://www.sabiosciences.com/pcrarraydataanalysis.php).

## 3. Results

**Figure 2:**
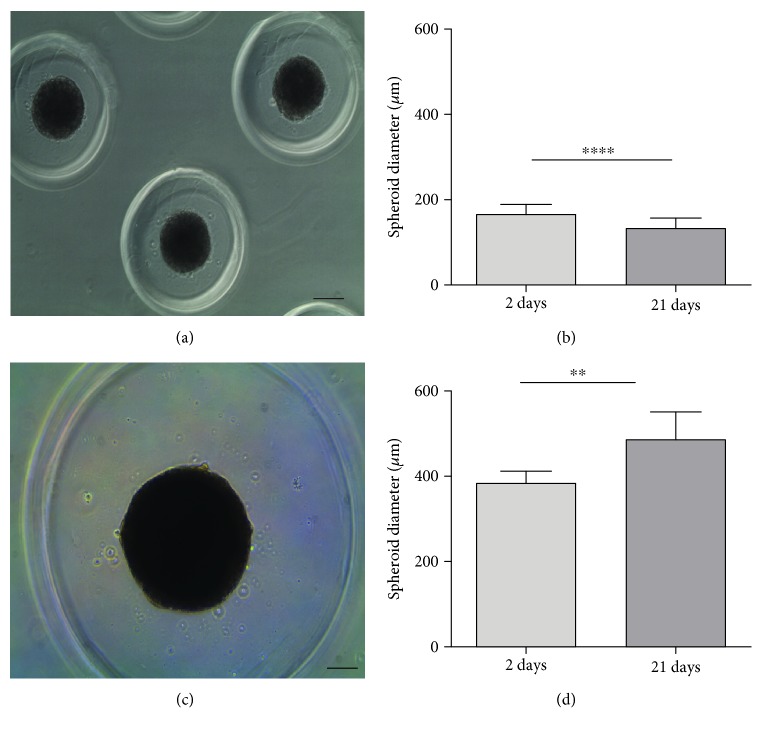
The CPC spheroids formed in the micromolded nonadhesive hydrogel with 800 *μ*m diameter in each circular recesses showed stability in their diameter along 21 days of cell spheroid culture. (a, c) Representative images of CPC spheroids formed in micromolded nonadhesive hydrogel with 300 *μ*m diameter (a) and with 800 *μ*m diameter in each circular recesses (c). Phase contrast microscopy. Bar size: 100 *μ*m. (b, d) The graphs represent the mean ± standard error of the spheroid diameter in micromolded nonadhesive hydrogel with 300 *μ*m diameter (b) and with 800 *μ*m diameter in each circular recesses (d). Light gray bar represents 2 days of cell spheroid culture. Dark gray bar represents 21 days of cell spheroid culture. ^∗∗∗∗^*p* < 0.0001; ^∗∗^*p* < 0.01.

CPC spheroids with homogenous shape (Figures 2(a) and 2(c)) and size (Figures 2(b) and 2(d)) were produced by both nonadhesive hydrogel with 300 *μ*m or 800 *μ*m diameter in each circular recesses. Only 1 spheroid per circular recesses was observed. As expected, the micromolded nonadhesive hydrogel with 300 *μ*m diameter formed CPC spheroids with a reduced diameter, approximately twice smaller compared to 800 *μ*m circular recess counterpart (Figure 2(b) and 2(d)). After 21 days of cell culture, CPC spheroids formed by the micromolded nonadhesive hydrogel with 300 *μ*m diameter in each recesses have reduced their diameter. Conversely, the CPC spheroids formed by the micromolded nonadhesive hydrogel with 800 *μ*m circular recesses have increased their diameter in about 100 micrometers (Figure 2(d)).

**Figure 3:**
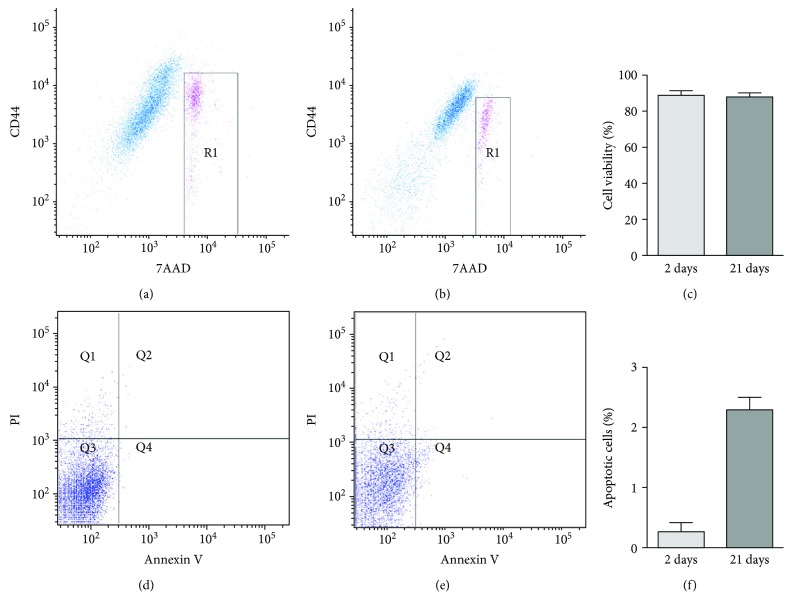
The CPC spheroids formed in the micromolded nonadhesive hydrogel with 800 *μ*m diameter in each circular recesses showed a high percentage of viability after spontaneous chondrogenesis. (a, b) Dot-plot graphs showing CPC positive for only CD44 in blue and double positive for CD44 and 7AAD in pink, after digestion of spheroids cultivated for 2 (a) and 21 (b) days. The viable cells were identified by 7AAD exclusion, outside the region R1. (c) The graph represents the percentage of viable cells in three different samples (*n* = 3). (d, e) Dot-plot graphs of annexin V and PI evaluation showing CPC positive for annexin V in Q2 and Q4, after digestion of spheroids cultivated for 2 (d) and 21 (e) days. (f) The graph represents the percentage of apoptotic cells (annexin V positive cells) in three different samples (*n* = 3). 7AAD: 7-actinomycin D; CD: cell cluster of differentiation; PI: propidium iodide.

Due to the diameter stability of CPC spheroids exclusively found in nonadhesive hydrogel with 800 *μ*m diameter in each circular recesses, all the subsequent experiments were carried out using this type of molded hydrogel. The viability assay performed by flow cytometry revealed a high percentage of viable cells in the digested mass of CPC spheroids after spheroid compaction (2-day culture) and spontaneous chondrogenesis (21-day culture) (Figures 3(a)–3(c)). The CPC cells were identified in digested mass of CPC spheroids by CD44 positivity (Figures 3(a) and 3(b)). Furthermore, apoptotic cells (annexin V positive cells) were rare (Figures 3(d)–3(f)).

**Figure 4:**
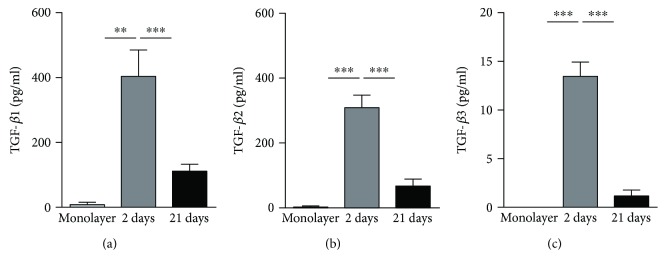
The CPC spheroids formed in the micromolded nonadhesive hydrogel with 800 *μ*m diameter in each circular recesses showed an increase in TGF-*β*1, TGF-*β*2, and TGF-*β*3 synthesis comparing to CPCs monolayer. The CPC spheroids maintained for 2 days (spheroid compaction) in cell spheroid culture showed the highest synthesis of TGF-*β*1 (a), TGF-*β*2 (b), and TGF-*β*3 (c) comparing to the CPC spheroids maintained for 21 days (spontaneous chondrogenesis) and to monolayer (^∗∗^*p* < 0.01, ^∗∗∗^*p* < 0.005). TGF-beta: transforming growth factor-beta.

The dosage of the three isoforms of TGF-*β* was performed since this superfamily initiate the chondrogenic differentiation. After spheroid compaction (2-day culture), the synthesis of TGF-*β*1, TGF-*β*2, and TGF-*β*3 was significantly higher compared to those observed in monolayer (Figures 4(a)–4(c)) (*p* < 0.005). The levels of the three TGF-*β* proteins evaluated in this study presented a decrease after spontaneous chondrogenesis (21-day culture) with TGF-*β*3 exhibiting the lowest levels in cell spheroid culture (Figure 4(b)). Despite the decrease observed in the synthesis of TGF-*β*1 after 21 days of cell culture, the levels of TGF-*β*1 is still higher compared to TGF-*β*3 levels (Figures 4(a) and 4(c)).

**Figure 5:**
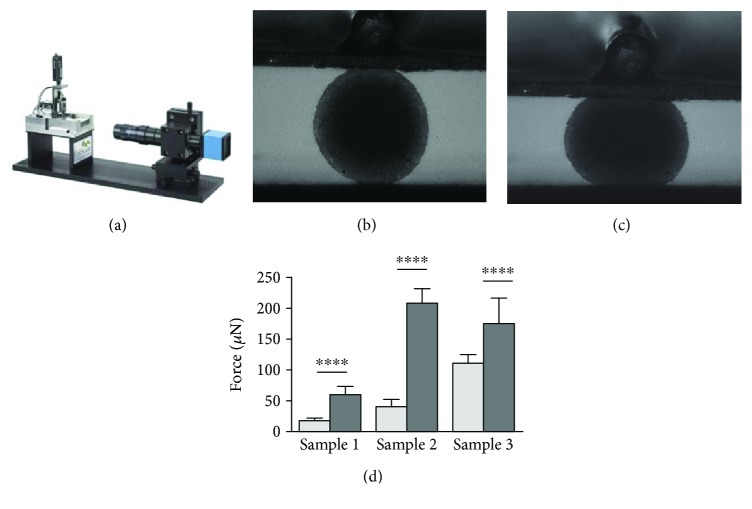
The CPC spheroids formed in the hydrogel with 800 *μ*m diameter in each circular recesses show enhanced resistance to compression after spontaneous chondrogenesis (21-day culture) compared to those cultured for 2 days (spheroids compaction). (a) Equipment used to perform the loading tests. (b, c) One CPC spheroid between 2 plates before and after compression, respectively. Note spheroid deformation of 25% of the original diameter in (c). Five compression cycles were performed, with a load phase duration of 20 seconds and a recovery phase of 10 seconds. (d) Data collected from 3 spheroids of three different samples after spheroid compaction (2 days, light gray column) and after spontaneous chondrogenesis (21 days, dark gray column) are expressed as mean ± SD. ^∗∗∗∗^*p* < 0.0001.

As a typical functional assay for cartilage tissue, compressive tests were performed to compare the biomechanical properties of spheroids after spontaneous chondrogenesis (21-day culture) with those after compaction (2-day culture). CPC spheroids cultured for 21 days showed an increase in their force (*μ*N) up to 4 times compared with those cultured for 2 days (Figure 5(d)), leading to a maximum deformation of 25% of their original diameter (Figures 5(b) and 5(c)).

**Figure 6:**
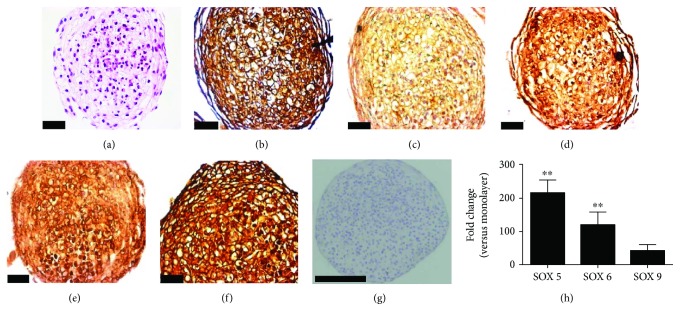
The CPC spheroids after spontaneous chondrogenesis are strongly positive for typical markers of cartilage tissue. (a) Hematoxylin and Eosin staining revealed rather rounded cells inside the spheroid. (b) N-cadherin is strongly present; (c–f) extracellular matrix typical markers of cartilage are shown throughout the spheroid area; (c) collagen type II; (d) collagen type VI; (e) aggrecan; (f) chondroitin sulfate. Bar size: 20 *μ*m. (g) Control of nonspecific binding of secondary antibody (immune reaction carried out in the absence of the primary antibody). All reactions were done in the same moment. Bar size: 100 *μ*m; *μ*m: micrometers. (h) qPCR analysis of CPC spheroids showing upregulation of the SOX5 and SOX6 genes comparing to monolayer after 21 days of cell culture (^∗∗^*p* < 0.01). The expression of SOX9 gene was not statistically significant (*p* = 0.05293). Standard errors are shown.

Histological sections stained by Hematoxylin and Eosin showed a fibroblastic morphology in the external layer of CPC spheroids in contrast to a rounded shape inside (Figure 6(a)). The CPC spheroids were completely positive for N-cadherin (Figure 6(b)). Considering the extracellular matrix components, CPC spheroids also were positive for collagen type II and type VI (Figures 6(c) and 6(d)) and for the sulfated glycosaminoglycan aggrecan and chondroitin sulfate (Figures 6(e) and 6(f)) after spontaneous chondrogenesis (21-day culture).

The expression levels of SOX genes involved in the chondrogenic process were compared between cells cultivated as monolayer and as spheroids. The SOX5 and 6 genes were upregulated after spontaneous chondrogenesis (21-day culture) (*p* < 0.001). As shown in Figure 6(h), SOX5 and SOX6 genes were more than a hundred times more expressed in spheroids than in the monolayer counterpart used as control. Although SOX9 presented a fold-change higher than 30 times in spheroid samples compared to controls (monolayer), this difference in expression levels was not statistically significant (*p* = 0.05293).

**Figure 7:**
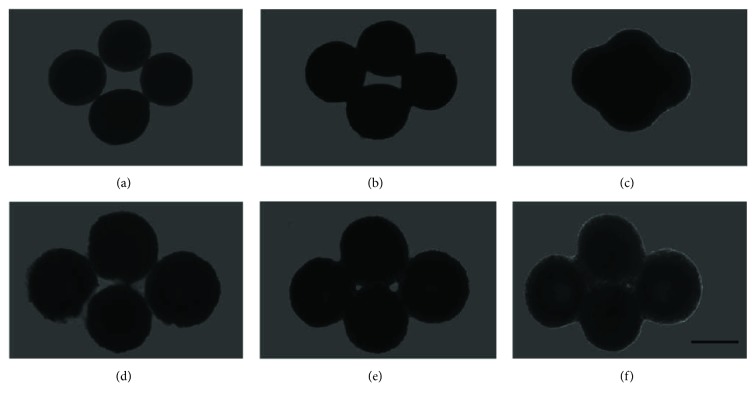
The CPC spheroids retained their fusion capacity even after spontaneous chondrogenesis. The CPC spheroids maintained for 2 days (a–c) and 21 days (d–f) in cell spheroid culture retained their fusion capacity. The CPC spheroids in close contact after 1 hour (a, d), 1 day (b, e) and 7 days (c, f). Note a complete fusion in c. Phase contrast microscopy. Bar size: 400 *μ*m.

Comparing to CPC spheroids after compaction (2-day culture), spheroids after spontaneous chondrogenesis (21-day culture) showed a reduced capacity of fusion. The spheroids were plated in close contact and after 1 hour, 1 and 7 days, a progressive increase in such proximity was noticed (Figures 7(a)–7(f)). A complete fusion was observed after 7 days in only CPC spheroids after compaction (2-day culture) (Figure 7(c)).

## 4. Discussion

The scaffold-free 3D cartilage constructs from human CPCs from nasal septum were successfully low-cost produced using the micromolded nonadhesive hydrogel technology. The spheroids showed homogeneity in their sizes and shapes, crucial for an efficient delivery of scaffold-free cartilage constructs for regenerative medicine approaches [22]. The CPC spheroids were established based on the self-assembling principle and recapitulated the chondrogenic differentiation pathway evaluated by TGF-*β*1, TGF-*β*2, and TGF-*β*3 synthesis, increasing force module, presented cartilage extracellular matrix components, and overexpressed SOX5 and SOX6 genes. In addition, we showed the *in vitro* capacity of CPC spheroids close to each other and even to fuse that could be interesting for successful *in vivo* implantation and long-term retention.

The commonly scaffold-free 3D technologies employed for cartilage tissue engineering are based on pellet culture [20], hanging-drop [23], and 96-well plate [10, 24], with the last two methodologies responsible for spheroid formation. Hanging-drop is a scalable technology; however, spheroids can be maintained in culture only for few days [23]. Recently, micromolded hydrogels have emerged as an alternative for spheroid culture due to the automation capacity and long-term culture for differentiation assays [25]. In contrast to 96-well culture plate, the micromolded nonadhesive hydrogel allowing to seed cell suspension with single pipetting, reducing substantially technical errors reflected in a homogeneity in spheroids size and shape. Besides, compared to 96-well culture plate, it is possible to obtain 10 times more spheroids per plate [3].

In this study, CPC spheroids showed homogeneity and stability in their size and shapes only in the micromolded nonadhesive hydrogel with 800 *μ*m in each recesses. The difference between the two types of micromolded used in this study dwells on the diameter of each circular recesses, since both of them present 800 *μ*m depth in each recesses (http://www.microtissues.com/3d-cell-culture-products.html). We hypothesize that the diameter found in micromolded with 300 *μ*m in each recesses was not large enough to allow a precise self-assembly process in the entire cell population confined in each recesses.

The CPC spheroids established with the 800 *μ*m micromold presented the diameter ranging from 486 *μ*m ± 65. Usually, the diameter of CPC spheroids is smaller than that observed for the recesses due to the compaction phenomenon driven by adhesion molecules, mainly N-cadherins, similar to condensation phase in developing embryo [5, 9]. In this study, the compaction phenomenon was observed after 2 days of CPC spheroid culture.

In addition to homogeneity and stability in size and shape along spheroid culture, the CPC spheroids produced with micromolded with 800 *μ*m in each recesses showed a high percentage (88.1 ± 2.1) of viable cells even after spontaneous chondrogenesis (21-day culture). A high percentage of viability is mandatory for cell differentiation and subsequent *in vivo* implantation of the scaffold-free 3D construct [26]. Viable cells were quantified by flow cytometry after the digestion of spheroids [21]. Besides 7AAD staining exclusion strategy (death cells), we used the surface marker CD44 to identify CPC cells [20] in the digested mass of cell spheroids. Only 7AAD-negative cells and CD44-positive cells were considered in our analyses. More importantly, cells in the core of spheroids can be dead by apoptosis. Recently, Murphy et al. showed considerable rate of apoptosis only in mesenchymal stem cell spheroids ranging from 700 *μ*m [27]. In our study, apoptosis was a rare event even after spontaneous chondrogenesis (21-day culture).

After compaction phenomenon (2-day culture), the synthesis of TGF-*β*1, TGF-*β*2, and TGF-*β*3 by the CPC spheroids increases considerably comparing to monolayer. This increase may be related to the first step of chondrogenesis, known as the condensation phenomenon [5], similar to compaction phenomenon observed in spheroids driven by cadherins. Interestingly, the synthesis of all TGF-*β* isoforms decreases after spontaneous chondrogenesis (21-day culture). Our result supporting the hypothesis that TGF-*β* is crucial for the first phase of chondrogenesis [28].

The TGF-*β*1 and TGF-*β*3 isoforms are attributed to chondrogenesis process, with the isoform *β*3 with strong *in vitro* evidence for successful chondrogenesis in human mesenchymal stem cells [28]. In chondrocytes, TGF-*β*1 induces extracellular matrix synthesis [29]. Because our source of cells represents a progenitor cell derived from cartilage, it is reasonable to attribute the high synthesis of TGF-*β*1 to the extracellular matrix synthesis evaluated at 21 days of CPC spheroid culture. Besides, TGF-*β*1 synthesis supports our hypothesis of spontaneous chondrogenesis of CPC spheroids. Furthermore, at 21-day culture, the CPC spheroids showed an increase in resistance to compression over time of spheroid culture as part of the successfully chondrogenic differentiation process. Our CPC spheroids have supported a deformation of 25% of their original diameter, reaching a maximum force of approximately 200 *μ*N, possibly due to their extracellular matrix components—collagen type II and aggrecan [30].

Immunohistochemistry analysis revealed a mature extracellular matrix of cartilage tissue after spontaneous chondrogenesis (21-day culture). We noticed a strong and homogeneous staining for collagen type II, collagen type VI, aggrecan, and chondroitin sulfate, all of them are typical markers of cartilage tissue. Curiously, the positivity for N-cadherin was maintained until 21 days of CPC spheroid culture. During embryogenesis, this adhesion molecule is crucial for the mesenchymal condensation, the first step of chondrogenic differentiation [5].

Some studies have shown that N-cadherin cleavage is necessary for the progress of chondrogenesis [31]. Nevertheless, other studies showed a positive influence of N-cadherin not only in the condensation step but also in the extracellular matrix synthesis after *in vivo* implantation [32]. Besides cell-extracellular matrix adhesion, the cadherin-mediated adhesion is also involved in mechanotransduction [33]. In this study, the positivity for N-cadherin maintained after spontaneous chondrogenesis (21-day culture) may take account to the increase in resistance to compression over time of spheroid culture. Also at 21-day culture, the huge overexpression of SOX trio, especially SOX5 and SOX6, supports the successful differentiation of CPC spheroids towards chondrogenic pathway [8]. Likewise, just like N-cadherin, the SOX trio acts together to increase the expression of proteins related to extracellular matrix synthesis, such as COL2A1 [34], the main collagen of cartilage tissue.

As a preliminary step for *in vivo* assays, we have tested the ability of CPC spheroids to fuse with each other. Interestingly, despite the high content of extracellular matrix observed in CPC spheroids after spontaneous chondrogenesis (21-day culture), they were able to start to fuse with each other. However, the complete fusion was observed exclusively in CPC spheroids after compaction phenomenon (2-day culture). The capacity of fusion can be inversely correlated with the increase in resistance to compression over time of spheroid culture. Since spheroids follow the laws of fluid mechanics [35], it is reasonable to postulate that the increase in superficial tension can lead to a resistance in spheroids to fusion. On the other hand, this increase in superficial tension reflects a stability in spheroids and may indicate a tissue maturity. Although scaffold-free approaches seem to be well integrated with surrounding cartilage tissue, the integration boundary is still fragile [4]. The intrinsic capacity of fusion from CPC spheroids after compaction phenomenon (2-day culture) could be crucial for a long-term retention in implantation site.

The micromolded nonadhesive hydrogel is a promise technology for automatized biofabrication scaffold-free 3D constructs. In this study, spheroids of CPCs were responsible for successful low-cost cartilage scaffold-free tissue engineering without exogenous stimulus (spontaneous differentiation), supporting the use of CPC spheroids for tissue engineering and regenerative medicine approaches. Our next step will be delivery CPC spheroids in a preclinical *in vivo* assay. Furthermore, the development of automation protocols is in progress, aiming the scaling of cell spheroid culture and scaffold-free 3D cartilage large construct biofabrication by bioprinting approaches [36].

## 5. Conclusion

The fabrication of 3D scaffold-free cartilage constructs using micromolded nonadhesive hydrogel was responsible for cartilage constructs with homogeneous size and shape and high cell viability together with the possibility of scaling cell spheroid culture using automation. To the best of our knowledge, this is the first time in the scientific literature that spheroids of CPCs from human nasal septum were produced using micromolded nonadhesive hydrogel, achieving a successful cartilage low-cost scaffold-free tissue engineering without exogenous stimulus. Delivering CPC spheroids introduce a great promise to improve retention in cartilage tissue implantation site.
